# PLGA-BMP-2 and PLA-17β-Estradiol Microspheres Reinforcing a Composite Hydrogel for Bone Regeneration in Osteoporosis

**DOI:** 10.3390/pharmaceutics11120648

**Published:** 2019-12-03

**Authors:** Patricia García-García, Ricardo Reyes, Elisabet Segredo-Morales, Edgar Pérez-Herrero, Araceli Delgado, Carmen Évora

**Affiliations:** 1 Department of Chemical Engineering and Pharmaceutical Technology, University of La Laguna, 38206 La Laguna, Spainesegredm@ull.edu.es (E.S.-M.); eperezhe@ull.edu.es (E.P.-H.); 2 Institute of Biomedical Technologies (ITB), University of La Laguna, 38206 La Laguna, Spain; rreyesro@ull.edu.es; 3 Department of Biochemistry, Microbiology, Cell Biology and Genetics, University of La Laguna, 38206 La Laguna, Spain

**Keywords:** BMP-2-microspheres, hydrogel system, 17-βestradiol release, bone regeneration, osteoporosis, poly-lactide-*co*-glycolide, polylactic acid

## Abstract

The controlled release of active substances—bone morphogenetic protein 2 (BMP-2) and 17β-estradiol—is one of the main aspects to be taken into account to successfully regenerate a tissue defect. In this study, BMP-2- and 17β-estradiol-loaded microspheres were combined in a sandwich-like system formed by a hydrogel core composed of chitosan (CHT) collagen, 2-hidroxipropil γ-ciclodextrin (HP-γ-CD), nanoparticles of hydroxyapatite (nano-HAP), and an electrospun mesh shell prepared with two external electrospinning films for the regeneration of a critical bone defect in osteoporotic rats. Microspheres were made with poly-lactide-*co*-glycolide (PLGA) to encapsulate BMP-2, whereas the different formulations of 17β-estradiol were prepared with poly-lactic acid (PLA) and PLGA. The in vitro and in vivo BMP-2 delivered from the system fitted a biphasic profile. Although the in vivo burst effect was higher than in vitro the second phases (lasted up to 6 weeks) were parallel, the release rate ranged between 55 and 70 ng/day. The in vitro release kinetics of the 17β-estradiol dissolved in the polymeric matrix of the microspheres depended on the partition coefficient. The 17β-estradiol was slowly released from the core system using an aqueous release medium (*D_eff_* = 5.58·10^−16^ ± 9.81·10^−17^m^2^s^−1^) and very fast in MeOH-water (50:50). The hydrogel core system was injectable, and approximately 83% of the loaded dose is uniformly discharged through a 20G needle. The system placed in the defect was easily adapted to the defect shape and after 12 weeks approximately 50% of the defect was refilled by new tissue. None differences were observed between the osteoporotic and non-osteoporotic groups. Despite the role of 17β-estradiol on the bone remodeling process, the obtained results in this study suggest that the observed regeneration was only due to the controlled rate released of BMP-2 from the PLGA microspheres.

## 1. Introduction

Regeneration of bone critical defects is still a challenge in the orthopedic field. Local treatment with bone morphogenetic protein (BMP-2) incorporated in different biomaterial scaffolds has demonstrated to be efficient to induce new bone formation for critical bone defect in several animal models. Nowadays, collagen sponges loaded with recombinant BMP-2 are clinically available as bone graft substitutes for the treatment of nonunion and critical-sized bone defects. Although BMP-2 is a potent osteogenic agent, a controlled release profile is required for safety and efficacy. Thus, the scaffold to fill the bone defect should not only be designed to act as support and guide for tissue growth, but also to control the release rate of active substances. Although many materials and structures have been proposed to construct these scaffolds, the control of the release rate has not always been taken into account. In fact, in some cases the protein is incorporated in the material by incubation and, unless a material-protein interaction occurs, a burst release at an early period would be expected. Consequently, a high dose of BMP-2 would be in blood circulation, leading to a high risk of side effects and, at the same time, a significant loss of the protein in the site of action. In addition, some authors showed that BMPs in these cases might also stimulate bone resorption due to the high dose of BMP-2 associated to uncontrolled release [1]. To minimize the osteoclastic effect of BMPs, some authors proposed the addition of an anti-catabolic agent. The most frequently studied combination has been BMP-2 with bisphosphonates as anti-resorption agents such as alendronate [2,3] and zolendronate [4]. The results indicated good bone regeneration with improved bone quality and mineralization in different localizations compared to BMP-2 alone [5,6].

Among the several natural polymeric scaffolds prepared for bone regeneration, chitosan is a biomaterial frequently used for this purpose. In a recent extensive review [7] based on chitosan (CHT) applied in bone tissue regeneration, different advantageous aspects were showed and discussed such as biocompatibility, capacity for BMPs sustained release, improvement of cell proliferation, and increase of in vitro and in vivo differentiation and mineralization. However, bone graft materials to simulate bone structure, e.g., collagen, the major ECM of bone tissue, and hydroxyapatite (HAP), a mineral component of the bone, have also been widely studied [6,8,9,10,11,12,13,14].

Although the aforementioned studies indicated positive results, the mentioned strategies applied in osteoporosis (OP) conditions have not always been effective [15]. According to recent reports, the prolonged subcutaneous administration of alendronate and the low level of estrogen in OP alters the evolution of calvarial bone repair due to estrogen, Transforming Growth Factor beta 1 (TGF-β1), and α-estrogen receptor (α-ER) interaction [16]. Menopausal women are the population most affected by this disease due to estrogen deficiency. Previous reports revealed that local implantation of scaffolds loaded with combinations of BMP-2 and 17β-estradiol formulated in microspheres of polylactic acid (PLA) or PLGA, in rat calvaria critical defects increased the bone repair in OP rats, but the new bone that refilled the defect was less mineralized compared to non-OP groups [17,18].

As some biomaterials may promote bone regeneration, controlled release of the active substances is required for efficient and safe bone regeneration [19,20]. In this study, we propose a loaded BMP-2 and 17β-estradiol sandwich-like system, comprising two polymeric external films and a core of a biocomposite hydrogel containing microspheres, to provide sustained release of active substances. The core system composed of CHT, collagen, HAP nanoparticles (nano-HAP), polyethylene glycol (PEG-400) and 2-Hidroxipropil γ-Ciclodextrin HP-γ-CD was previously characterized in terms of composition, rheological behavior and mass-transfer using RITC-dextran as macromolecule model and 17β-estradiol in microspheres [21]. In the present study, we aim to study the influence of the release rate of 17β-estradiol on the osteogenic effect induced by BMP-2 released from PLGA microspheres within core system after sandwich-like system implantation in a OP rats critical size defect. Therefore, 17β-estradiol was incorporated into the system in 3 forms: free and dispersed in the core, encapsulated in microspheres prepared with a mixture of PLA and PLGA dispersed within the hydrogel and lastly encapsulated in the PLGA films shell prepared by electrospinning technique.

## 2. Materials and Method

### 2.1. Materials

PLGA 75:25 (Resomer^®^ RG755-S), PLGA 50:50 (Resomer^®^ RG504), PLGA 85:15 (Resomer^®^ RG858-S), and PLA (Resomer^®^ RG203-S) were supplied by Evonic Industries (Darmstadt, Germany). Chitosan (Protasan^®^ UP-CL-213) was purchased from NovaMatrix (Sandvika, Norway). 2-Hidroxipropil γ-Ciclodextrin (CAVASOL^®^ W8 HP), was supplied by Wacker Chemical (Burghausen, Germany). The bovine collagen type I was purchased from CellSystems Biotechnologie (Vertrieb GmbH, Germany). Riboflavin (RB), Poly(ethylene glycol) 400, Poly(vinyl alcohol) (PVA, Mw 33–70 kDa; 87–90% hydrolyzed), 17β-estradiol, and all the other reagents were purchased from Sigma-Aldrich, (St. Louis, MO, USA). The recombinant human bone morphogenetic protein 2 (BMP-2) was bought from Biomedal Life Sciences (Sevilla, Spain). Citrate-coated carbonated apatite nanoparticles (nano-HAP) were kindly donated (Jaime Gómez-Morales, PhD, Laboratory of Crystallographic Studies, CSIC, Granada, Spain).

### 2.2. Microspheres Preparation and Characterization

The BMP-2 microspheres were prepared by the double emulsion method (w/o/w) previously described [22]. Briefly, 200 µL of an aqueous solution (0.2% PVA) of BMP-2 (260µg) was emulsified with 1 mL of a PLGA mixture (150 mg) of RG504 and RG858 [4:1] in methylene chloride (DCM) by vortexing 1 min (position 10, Genie^®^ Industries 2, Sciencies Industries Inc. USA). Then, this emulsion was poured into 10 mL of 0.2% PVA solution vortexed 15 s, then poured into 100 mL of 0.1% PVA and kept under magnetic stirring for 1 h for solvent evaporation.

The 17β-estradiol microspheres were prepared by a modified solvent evaporation method previously described [17]. Briefly a mixture of 17β-estradiol (4mg), PLA-S RG203-S (160 mg) and PLGA RG858 (40 mg) dissolved in 0.6 mL of DCM:Methanol (DCM:MeOH) (80:20) was emulsified with 4 mL of 1% PVA aqueous solution by vortexing 1 min (position 10), and then added to 100 mL of 0.16% PVA solution, under magnetic stirring for organic solvent evaporation (1 h).

Both type of microspheres were collected by filtration (Pall Corporation, pore size 45 µm, Sigma-Aldrich, USA), lyophilized, and stored at 4 °C until use.

Microspheres were characterized in terms of size (Mastersizer 2000, Malver Instruments, Malvern, UK) and morphology (SEM, Jeol JSM-6300, Tokyo, Japan). To determine the BMP-2 encapsulation efficiency and to carry out the BMP-2 release assays, some batches were prepared with ^125^IBMP-2. The BMP-2 was labeled with ^125^INa (Perkin-Elmer) by the iodogen method [23]. The content of 17β-estradiol in the microspheres was determined spectrophotometrically at λ = 280 nm previous dissolution in a mix of DCM:MeOH (80:20).

To determine the solubility of 17β-estradiol in the polymer matrix of the microspheres, differential scanning calorimetry (DSC 025, TA Instruments, New Castle, DE, USA) was performed. 17β-estradiol and lyophilized microspheres were analyzed after drying in an oven at 37 °C overnight. In addition, samples of polymer blends (RG 203-S and RG 858, 4:1) and samples of the polymer blend with excess 17β-estradiol (8.5%) were dissolved in DCM:MeOH (80:20) and maintained in a hood for 24 h. Then, samples were placed 48 h more in a vacuum desiccator to complete the evaporation of the organic solvent. The analysis of all samples was performed with the same thermal program in two thermal cycles under a nitrogen atmosphere (50 mL/min). In the first cycle, temperature was increased to 40 °C (10 °C/min) and then cooled to −20 °C (5 °C/min) to avoid possible water interference. Once the samples were stabilized, they underwent a final heating cycle from −20 °C to 270 °C (10 °C/min).

### 2.3. Fabrication and Characterization of the Film

The film was fabricated by a previously described electrospinning method [24]. Briefly, 7 mg of 17β-estradiol and 300 mg of a mixture of PLGAs, RG755-S and RG858 [4:1] were dissolved in 2 mL of hexafluoroisopropanol (Sigma-Aldrich, Steinheim, Germany) and electrospun at 7kV; flow rate of 3.0 mL/h and 10 cm of distance from the collector.

The film quality was checked in terms of porosity, thickness and fiber diameter using helium pycnometer (AccuPyc 1330, Micromeritics, Norcross, GA, USA), stereo microscopy (Leica M205C, Leica Las, v3 sofware), and SEM (Jeol, JSM-6300, Tokyo, Japan), respectively.

### 2.4. Core System Preparation and Characterization

To prepare the core system, approximately 20 mg of microspheres were dispersed in 50 µL of the hydrogel composed by a mixture of collagen type I (5 mg/mL), HP-γ-CD (34 mg/mL), RB (0.4 mg/mL), CHT (5 mg/mL), PEG-400 (150 mg/mL), and 5 mg of nano-HAP. Then, the hydrogel was cross-linked with 5% *w*/*w* TPP sterile aqueous solution (0.5 µL/µL of hydrogel) and visible light blue at 468 nm (Dental device) for 3 min [21]. The dose of BMP-2 was 6 µg in microspheres and the total 17β-estradiol dose was 200 µg in three different forms: electrospun films, microspheres, or dispersed into the gel.

The quality of the core system was checked according to its rheological characteristics and porosity previously described [21]. In addition, water uptake and mass loss assays were carried out by incubation of aliquots of 300 µL of the core system in 5 mL of sterile MilliQ water (37 °C) under orbital agitation (25 rpm). At specific times, six samples were withdrawn, we then removed excess water, weighed, and freeze-dried the samples. Then, three samples were visualized by SEM (Jeol JSM-6300) to see the evolution of the internal structure after incubation. The other three samples were used to record the dried weight and calculate the percentage of mass loss and water uptake, applying Equations (1) and (2), respectively, where *W*_0_ is the initial weight of the sample and *W_w_* and *W_d_* are the weights of the wet and dried sample, respectively, at the different times tested.

(1) $$Mass\;loss\left(\%\right)=\frac{\left(W_{0}-W_{d}\right)}{W_{0}}\;\times \;100$$

(2) $$Water\;uptake\left(\%\right)=\frac{\left(W_{w}-W_{d}\right)}{W_{d}}\;\times \;100$$

To test the syringeability of the core system two syringes of 1 mL were loaded with a suspension of microspheres of 17β-estradiol in the hydrogel, up to 0.5 mL. Then, 4 doses of 50 µL each were unloaded from both syringes assayed for fluidity through a 20G needle and dose uniformity. For this, the discharged samples were lyophilized, and the 17β-estradiol content evaluated by spectrophotometry at 280 nm, after dissolution in DCM: MeOH (80:20).

### 2.5. In Vitro Release Assays

BMP-2 in vitro release assays were carried out by incubating an amount of ^125^I-BMP-2 microspheres and an amount of core system with the same amount of ^125^I-BMP-2 microspheres in sterile MilliQ water at 37 °C and 25 rpm. The amount of BMP-2 released was calculated by measuring the radioactivity of supernatant samples with a gamma counter (Cobra^®^ II, Packard).

The in vitro release of 17β-estradiol from the different formulations (dispersed in the core system, microspheres, microspheres incorporated to the core system, and electrospun films) was carried out at 37 °C and 25 rpm using two release media: an aqueous solution of sodium lauryl sulfate (SLS) 1% [25] and MeOH:water (50:50) [26,27]. The released 17β-estradiol was measured in the supernatant using the spectrophotometric method. The effective diffusion coefficient, *D_eff_* in the matrix of the microspheres and the mass transfer coefficient of the drug in the boundary layer *h*, were calculated according to the non-steady-state Fick law, as previously described in detail [21]. Whether or not the released fraction of 17β-estradiol from the microspheres dispersed in the core system was analyzed, and Equations (3)–(6) were applied for *D_eff_* and *h* calculation.

(3) $$\frac{M_{t}}{M_{\infty }}=1-\overset{\infty }{\underset{n=1}{\sum }}\frac{6\;L^{2}}{\beta_{n}^{2}\;\left(\beta_{n}^{2}+L^{2}-L\right)}exp\left(-\frac{\beta_{n}^{2}}{R^{2}}\;D_{eff}\;t\right)$$

where *M_t_* and *M_∞_* are the total mass of drug released to the media at time *t* and at the end of the experiment, respectively. The *β_n_s* are the infinite roots (eigenvalues) of the Equation (4):

(4) $$\beta_{n}\cot\beta_{n}+L-1=0$$

*L* is the dimensionless mass transfer Biot number Equation (5):

(5) $$L=\frac{h\;R}{D_{eff}}$$

For large values of *L*, the roots of Equation (4) are multiples of the number *pi* and Equation (3) can be simplified in the Equation (6), that is, a simplified solution of non-steady-state Fick law:

(6) $$\frac{M_{t}}{M_{\infty }}=1-\frac{6}{\pi^{2}}\overset{\infty }{\underset{n=1}{\sum }}\frac{1}{n^{2}}exp\left(-\frac{n^{2}\;\pi^{2}}{R^{2}}\;D_{eff}\;t\right)$$

As stated previously, to minimize the residual sum of squares, “genetic algorithms” already implanted in R software (R Foundation for Statistical Computing, version 3.6.1., 2019, Vienna, Austria) were used [21].

### 2.6. Animal Experiments

All animal experiments were carried out in conformity with the European Directive (2010/63UE) on Care and Use in Experimental Procedures. Furthermore, the animal protocols were approved on 5 November 2014 by the Ethics Committee for Animal Cares of the University of La Laguna (CEIBA) with identification code CEIBA2014-0128. All surgical procedures were made under aseptic conditions.

#### 2.6.1. Animal Models

Forty female adult Sprague-Dawley rats approximately 12 weeks old, weighing 200–250 g, were divided in 4 groups of 10 each. The experimental osteoporosis was induced in 3 groups by three different protocols, OVX, chronic administration of DEX and OD. The forth group was the sham, non-osteoporotic control group (non-OP). The bilateral ovariectomy was carried out under isoflurane anesthesia, via dorsal approach to the animals of OVX and OD groups. Analgesia consisted in buprenorphine administered subcutaneously (0.05 mg/kg) before surgery and paracetamol (70 mg/100mL) in the water, for 3 days post-surgery. The DEX group received 0.3 mg/kg body weight of dexamethasone-21-isonicotinate (Deyanil retard, Fatro Ibérica, Barcelona, Spain) administered subcutaneously once ievery two weeks [28] up to the time of euthanasia. Then, two weeks after the ovariectomy, the rats of group OD were chronically treated with DEX as the DEX group. The 40 rats were sacrificed after 12 weeks and the calvaria and femurs were extracted to be histologically analyzed. The results of these analyzes were used to evaluate the 3 protocols tested to induce OP.

#### 2.6.2. Animals Groups

Fifty female Sprague-Dawley rats (12 weeks old), weighing 200–250 g, were divided into 2 groups of 25 each: OP and non-OP. The rats of the OP group were ovariectomized and the rats of the non-OP group underwent similar surgery but the ovaries were not resected. Twelve weeks post-surgery, 8 mm critical size cranial defects were created surgically with a trephine burr in the rats under isoflurane and the systems were placed into the defects [20]. Analgesia treatment was administrated.

Female rats were divided into 5 groups of 10 rats each—5 OP and 5 non-OP—and the applied regenerative treatment is reflected in Table 1. The implantation of the systems was carried out following a two steps procedure. First, a layer of film (bottom film), previously soaked in the blood produced during the surgery, was placed in the defect then 50 µL of the hydrogel mixed with the microspheres and partially cross-linked with UV light, was discharged. Second, the hydrogel was completely cross-linked by dripping 25 µL of sodium tripolyphosphate (TPP) forming the core system, after 5 min, a second layer of film (soaked in blood) was placed on the top, like a sandwich, and the wound was then closed.

#### 2.6.3. ^125^I-BMP-2 in Vivo Release Assay

The BMP-2 release kinetics was monitored periodically by measuring the remaining ^125^I-BMP-2 at the rat calvarial defect site (*n* = 5) using an external probe-type gamma counter (Captus ^®^, Capintec Inc., Ramsey, NJ, USA), as previously described and validated [29].

### 2.7. Rat Mesenchymal Stem Cells (rMSCs) Osteogenic Differentiation

The rMSCs were obtained by centrifugal isolation as previously described [30] from the bone marrow of the femur of OVX female Sprague-Dawley rats. Briefly, the cells were resuspended in high glucose DMEM (HyClone^®^ Utah) supplemented with 10% fetal bovine serum (Biowest, South America Origin), 1% penicillin–streptomycin (PAA, Pasching, Austria), and 2 mM l-Glutamine stable (Biowest, France) (Complete Culture Medium, CCM). Then, cells were cultured in flasks of 75 cm^2^ and subcultured by incubating at 37°C and 5% CO_2_. The culture medium was changed every 2–3 days.

To test the osteogenic differentiation, 50,000 cells (passage 2) in 20 μL of CCM were added over aliquots of 300 μL of the core system (hydrogel with microspheres) with and without nano-HAP and incubated at 37 °C and 5% CO_2_ for 1.5 h for cell adhesion. The homogeneous cell distribution was checked by light microscopy. Afterwards, 500 μL of CCM were added to each well, after 3 days incubation the medium was changed to CCM supplemented with 10 mM β-glycerol phosphate, 10^–7^ M dexamethasone and 50 μM ascorbate-2-phosphate. At 7, 14, and 21 days of culture, three wells of each time point were washed (2 times) with Hank’s balanced salt solution (HBSS 1x) and cooled at 4 °C. Then, 500 µL of 0.1 M buffer Tris-HCl, 0.1M NaCl, and 0.05 M MgCl_2_ (pH = 9.2–9.5) containing Nitro blue tetrazolium chloride (NBT, Roche Diagnostics, Mannheim, Germany) and 5-Bromo-4-chloro-3-indolyl phosphate (BCIP, Roche Diagnostics, Mannheim, Germany) were added and incubated at 37 °C and 5% CO_2_ under soft agitation for 1.5 h. Then, the NBT/BCIP was removed and the cells were fixed with a solution of 3.7–4% p-formaldehyde buffered to pH = 7.0 (Panreac^®^, Barcelona, Spain) during 30 min. After this, the formaldehyde was removed, and the wells were washed 3 times with HBBS 1x. Immediately after this, cells were visualized by stereo microscopy (Leica M205C, Leica Las, v3 software). In addition, samples were dehydrated in a graded series of ethanol before being embedded in Paraplast^®^ and microtome (Shandon Finesse 325, Thermo Fisher Scientific, Madrid, Spain) sections were observed by light microscopy (LEICA DM 4000B, Barcelona, Spain).

### 2.8. Histology, Immunohistochemical, and Histomorphometrically Evaluation

First, to check the osteoporotic-like condition, 12 weeks after the 12 rats undergone the different protocols were sacrificed and the femurs and calvaria were analyzed. The femurs and calvaria were fixed (4% paraformaldehyde solution), decalcified in Histofix^®^ Decalcifier (Panreac, Barcelona, Spain) and prepared for histological analysis as previously described [20,22].

Bone morphology was analyzed by hematoxylin–erythrosin staining. The histomorphometric analyze was carried out in femurs by measuring the following parameters; thickness of the cortical bone (Ct.Wi) and number (Tb.N), width (Tb.Wi) and separation (Tb.Sp) of the trabeculae in cancellous bone. In the calvaria bone, the histomorphometric analysis was carried out by measuring the following parameters, cortical bone thickness (CBT) and intercortical space thickness (IST) occupied by trabecular bone in transversal sections of calvaria.

To determine the capacity of the bone active substances, so as to regenerate the critical size defect practiced in the calvaria of the rats, samples of the 10 groups of 5 rats each were examined.

Samples were processed as previously described [22]. New bone formation was identified by hematoxylin–erythrosin staining. Bone mineralization was assessed with VOF trichrome stain, in which red and brown staining indicates advanced mineralization, whereas less mineralized, newly formed bone stains blue [31]. Sections were analyzed by light microscopy (LEICA DM 4000B, Barcelona, Spain). Computer-based image analysis software (Leica Q-win V3 Pro-Image Analysis System, Barcelona, Spain) was used to evaluate all sections. A region of interest (ROI) within the defect (50 mm^2^) for quantitative evaluation of new bone formation was defined. New bone formation was expressed as a percentage of repair with respect to the original defect area within the ROI. From the total bone repair, the areas of mature bone (MB) and immature bone (IB) were determined, and the MB/IB ratio for each experimental group as well as between non-osteoporotic and osteoporotic-like animals was calculated.

For immunohistochemical analysis, sections were deparaffined and rehydrated in Tris-buffered saline (TBS) (pH 7.4, 0.01 M Trizma base, 0.04 M Tris hydrochloride, 0.15 M NaCl), which was used for all further incubations and rinse steps. Sections were incubated in citrate buffer (pH 6) at 90°C for antigen retrieval, followed by incubation in 0.3% hydrogen peroxide in TBS buffer for 20 min. After a rinse step, sections were blocked with 2% FBS in TBS–0.2% Triton X-100 (blocking buffer). The indirect immunohistochemical procedure was carried out by incubating the sections with osteocalcin (OCN) polyclonal antiserum (1/100) (Millipore, Barcelona, Spain) in blocking buffer overnight at 4 °C. Sections were rinsed three times, then incubated with biotin-SP-conjugated donkey anti-rabbit F(ab) fragment (1/200) (Millipore, Barcelona, Spain) in blocking buffer for 1 h followed, after another rinse step, by incubation in peroxidase-conjugated streptavidin (1/300) (Millipore, Barcelona, Spain) for 1 h. Peroxidase activity was revealed in Tris–HCl buffer (0.05 M, pH 7.6) containing 0.05% of 3,3′-diaminobenzidine tetrahydrochloride (Sigma, Poole, UK) and 0.004% hydrogen peroxide. Reaction specificity was confirmed by replacing the specific antiserum with normal serum or by pre-adsorption of the specific antiserum with the corresponding antigen.

OCN staining was evaluated using computer-based image analysis software (ImageJ, NIH, Bethesda, MD, USA). OCN staining was measured by applying a fixed threshold to select for positive staining within the ROI. Positive pixel areas were divided by the total surface size (mm^2^) of the ROI. Values were normalized to those measured from blank scaffolds and are reported as relative staining intensities.

Statistical analysis was performed with SPSS.25 software. We compared the distinct treatments by means of a one-way analysis of variance (ANOVA) with a Tukey multiple comparison post-test. Significance was set at *p <* 0.05. Results are expressed as means ± SD.

## 3. Results

### 3.1. Sandwich-Like System Characterization

The characteristics of the microspheres, electrospun film, and core system are shown in Table 2.

**Figure 1:**
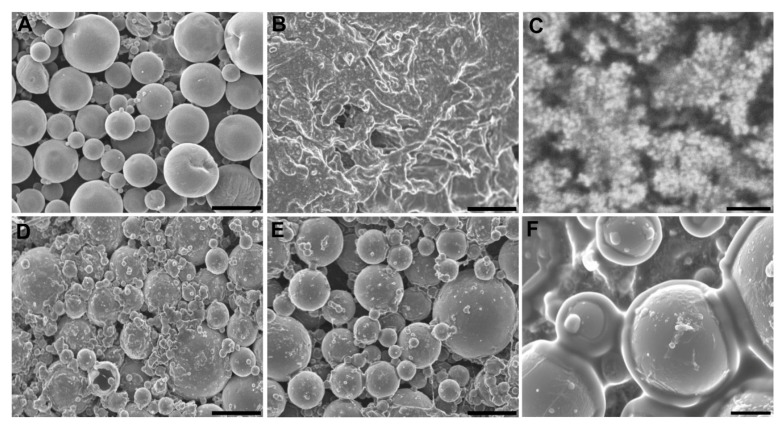
SEM images (**A**) microspheres, (**B**) hydrogel, (**C**) hydrogel high magnification detail, (**D**) core system freshly prepared, (**E**) internal structure of the core system after 4 weeks incubation in water at 37 °C and 25 rpm, and (**F**) high magnification detail of Figure 1E. Scale bars: (**A**–**E**) 100 µm, (**C**) 1 µm, (**F**) 20 µm.

**Figure 2:**
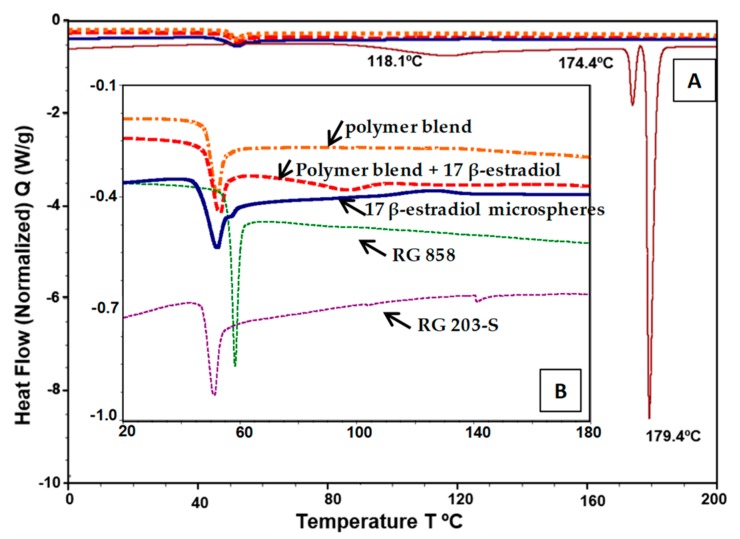
Differential scanning calorimetry thermograms. (**A**) Curve of pure 17β-estradiol. (**B**) Curves of PLA (RG203-S); PLGA (RG858), polymer blend (RG203-S:RG858, [4:1]), and polymer blend with 8.5% of 17β-estradiol, previously dissolved in DCM:MeOH (80:20) and the curve of the microspheres of 17β-estradiol (**B**).

SEM image of the microspheres is shown in Figure 1A and the differential scanning calorimetry thermograms of the 17β-estradiol microspheres components are plotted in Figure 2. The glass transition temperature (Tg) of the mixture of polymers RG 203-S and RG 858 [4: 1] was located at 52–58 °C, in the temperature range of the PLA (RG203-S) and PLGA (RG858). The DSC analysis of pure 17β-estradiol showed three endothermic peaks (Figure 2A), the first two at 118.1 °C and 174.4 °C, previously attributed to the partial and complete loss of hydrogen-bound water and reticular water, respectively. The third at 179.4 °C corresponds to the melting point [32]. This last peak, characteristic of the crystalline structure of 17β-estradiol, was not detected in the spectrum of the polymers and 17β-estradiol blend or in the thermogram of the microspheres (Figure 2B). These results indicated that 17β-estradiol was dissolved in the polymer by at least 8.5%.

The integrity of the system was assayed throughout the 4-week test duration. The SEM images of the internal structure of the hydrogel (Figure 1B,C), the freshly prepared core system (Figure 1D), and after 4 weeks incubation showed that the microspheres were homogeneously dispersed in the hydrogel and are trapped in the core system during incubation (Figure 1E,F). The core system absorbed a significant amount of water during the first days of incubation which was maintained over time. Contrarily, the system lost little mass: less than 20% during the first week and approximately 35% after 4 weeks (Table 2). In addition, the core system flew well through the 20 G needle and the average dose discharged was 83.5 ± 6% of the loaded dose.

### 3.2. Osteogenic Differentiation

**Figure 3:**
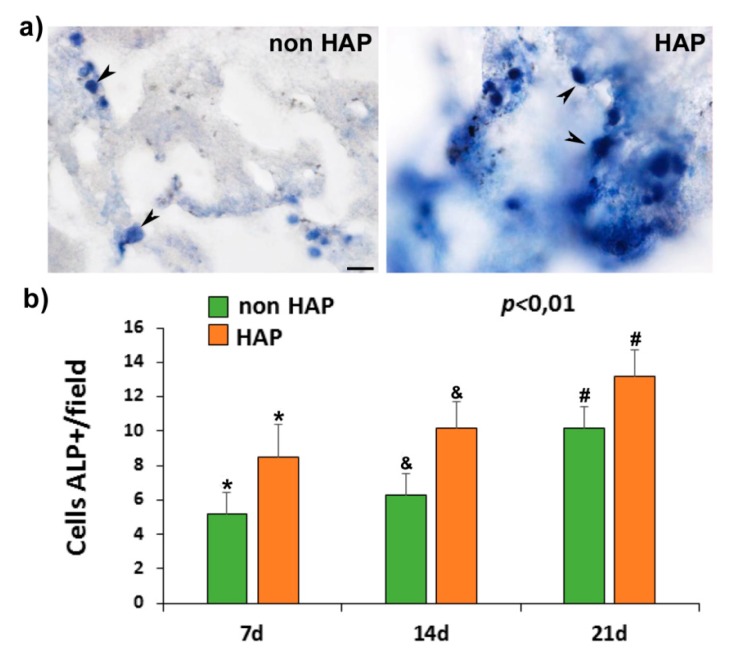
Alkaline phosphatase (ALP) activity in rMSCs cultures. (**a**) Representative images from hydrogels 20 days after cultured showing the AP-positive (ALP+) cells’ morphology (arrowheads) in hydrogel without (non-HAP) and with nanohydroxyapatite (HAP). (**b**) Graphic showing the number of APL+ cells/ microscopic field at different time points of analyses (7, 14, and 21 days) after culture in each system. Scale bar = 20 µm. The identical symbol on different bars indicates significant differences.

The alkaline phosphatase positive (ALP+) cell count, in the hydrogels pre-seeded with rMSCs and cultured in the osteogenic differentiation culture medium, showed a discrete and progressive increase in the number of cells between 7 and 21 days of culture in the hydrogels without and with nano-HAP; the number being significantly higher in those containing nano-HAP (Figure 3). Likewise, qualitatively, the cells presented, in the scaffolds with nano-HAP, greater intensity of color, suggesting greater ALP activity (Figure 3) than without nano-HAP.

### 3.3. Release Profiles of ^125^I-BMP-2 and 17β-Estradiol.

**Figure 4:**
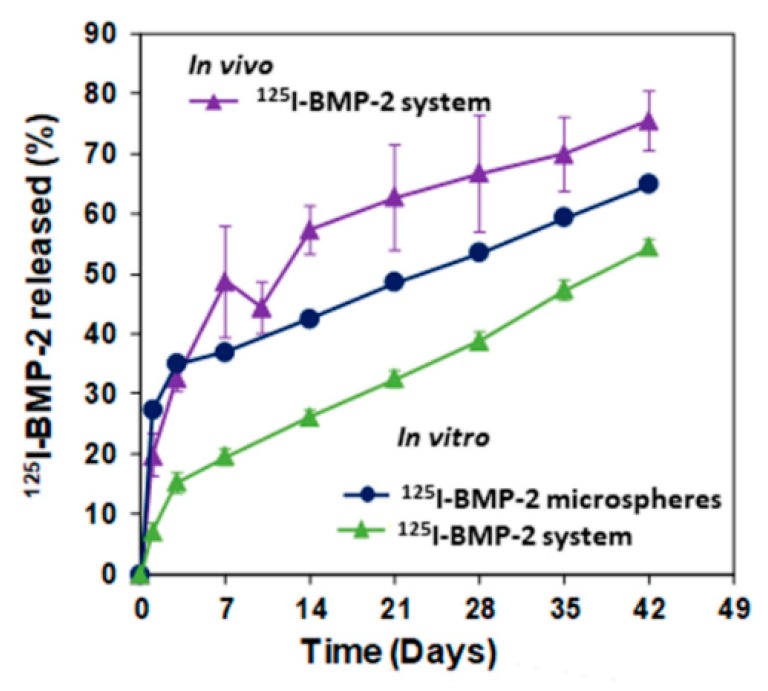
BMP-2 release assays. In vivo release profile of ^125^I-BMP-2 from PLGA-microspheres in the system after implantation in the rat calvaria defect (*n* = 5). In vitro release of ^125^I-BMP-2 (incubation in water at 37 °C and 25 rpm) from PLGA-microspheres and from the PLGA microspheres dispersed in the system.

Although the hydrogel provoked a strong reduction of the burst effect, the in vitro release of BMP-2 from the microspheres and from the core system showed a biphasic profile. During the first 24 h, approximately 7% of the protein was release from the core system versus 27% from the microspheres directly dispersed in the medium. Afterward, the release rate was kept in the range of 60 to 55 ng/day (Figure 4). The in vivo release profile was also biphasic, with a first phase that lasted up to 7 days whereas approximately 50% of the protein released. Then, the release rate was reduced to 70 ng/day, which is slightly higher than the in vitro rate.

**Figure 5:**
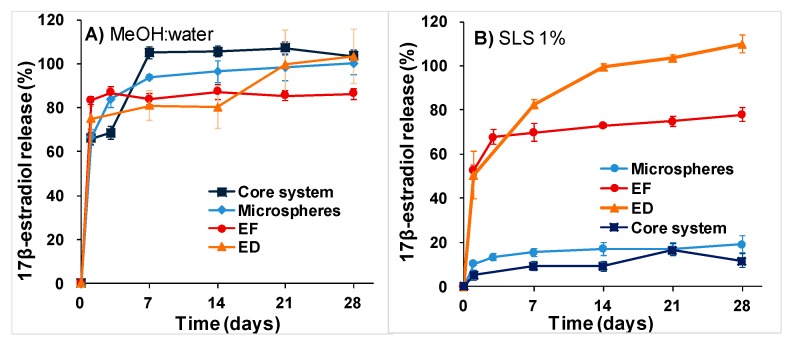
In vitro 17-β-estradiol release profiles in different release media at 37 °C and 25 rpm. (**A**) In MeOH:water (50:50). (**B**) In aqueous solution of SLS 1%. (ED) 17β-estradiol dispersed in the core system; (EF) 17β-estradiol in the electrospinned film; (microspheres) 17β-estradiol pre-encapsulated in microspheres and (core system) 17β-estradiol microspheres in the hydrogel.

The in vitro release rate of 17β-estradiol in the aqueous medium depended on the formulation (Figure 5B); 100% and 70% of the 17β-estradiol dispersed in the hydrogel and in the electrospun film were detected in the medium after 4 weeks incubation, respectively. The release profile was characterized by a high burst effect; approximately half of the dose was released during the first day. By contrast, the 17β-estradiol release rate was extremely slow from the microspheres alone and from the microspheres included in the hydrogel (core system). Both release profiles were similar: less than 20% was delivered in 4 weeks. However, in the MeOH: water (50:50) medium there were not differences in the transfer profiles. The presence of MeOH modifies the solubility of 17β-estradiol, showing a strong burst effect that varied in a range between 70 and 85% in the first 24 h (Figure 5A).

The estimated values of *D_eff_* and *h* for 17β-estradiol release in the different media are showed in Table 3. Although the value of *D_eff_* of 17β-estradiol in SLS was significantly lower compared to the MeOH:water (50:50), there were not differences for *h* regardless of the release media used. *h* is the contribution of hydrogel, as part of the boundary layer, to the whole mass transfer process, and the value of this coefficient should not change by varying the release media. The values of *R*^2^ (Table 3), together with the comparison of experimental and predicted values of the released fractions shows a good fit of the data to the proposed model for both release media.

### 3.4. Histology, Immunohistochemical, and Histomorphometrically Evaluation

#### 3.4.1. Osteoporotic Model

**Figure 6:**
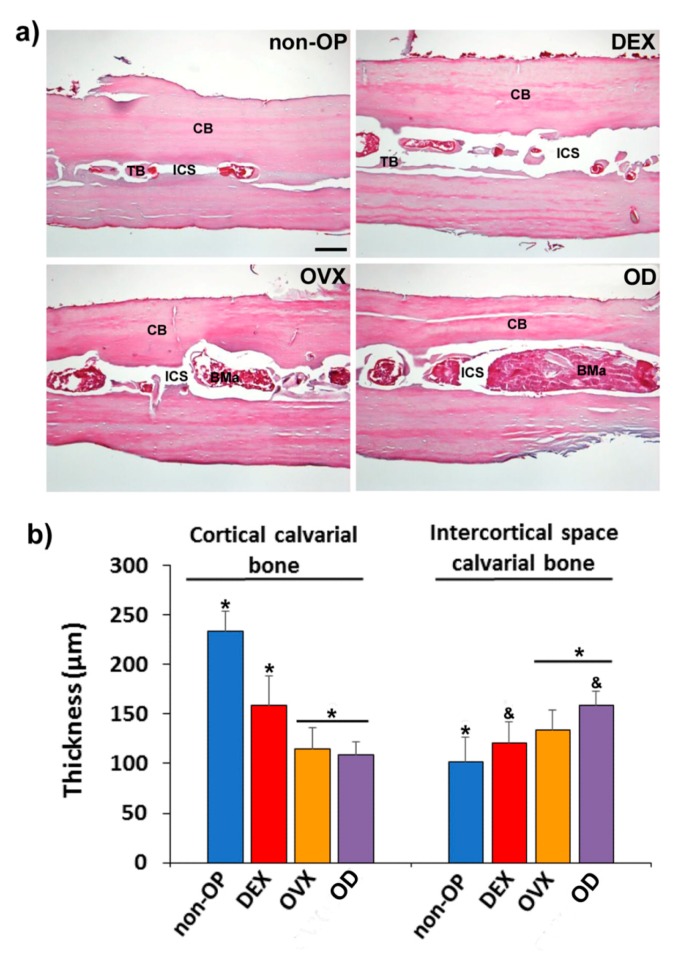
Validation of the OP model in calvarial bone. (**a**) Representative images in transversal section of calvaria in non-osteoporotic animals (non-OP) and in each of the different experimental models of osteoporosis tested showing the bone structure in each one. (**b**) Histomorphometric analysis of the cortical bone thickness and intercortical space thickness evaluated in calvaria in the different models of osteoporosis. CB: cortical bone; BMa: bone marrow; ICS: intercortical space; TB: trabecular bone. Scale bar = 100 µm. The identical symbol on different bars indicates significant differences.

The osteoporotic model was assessed in both long bone (femur) and flat bone (calvaria). The histological analysis of calvaria showed evident changes in the structure and microarchitecture of the bone among the different experimental groups. Although the non-OP animals showed a normal bone structure in cortical bone (CB) and trabecular bone (TB), in the intercortical space (ICS), for the different OP models (DEX, OVX, and OD (Figure 6a), it was observed a progressive decrease in cortical bone thickness (CBT) and an increase in the intercortical space thickness (IST), being the group OD the one that presented greater alteration of the tissue bone structure (Figure 6b).

**Figure 7:**
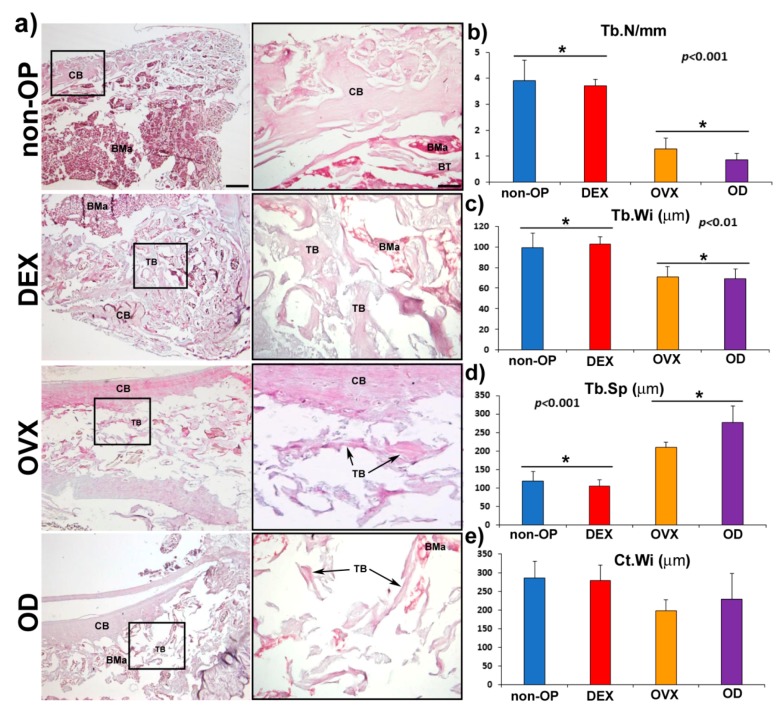
Validation of the OP model in long bone (femur). (**a**) The left column shows representative panoramic images in longitudinal section of rat femur in non-osteoporotic animals (non-OP) and in each of the different experimental models of osteoporosis tested. The right column shows high magnification images of the distal portion of the femur, showing differences in the microarchitecture of the bone in the different models. The column on the left shows detail of the boxed areas in which the structural characteristics of the compact and trabecular bone can be observed in each of the models. Histomorphometric analysis of the different parameters evaluated in femur in the different models of osteoporosis (**b**), Tb. N (mm), (**c**) Tb. Wi (µm), (**d**) Tb. Sp. (µm), and (**e**) Ct. Wi (µm). CB: cortical bone; TB: Trabecular bone; BMa: bone marrow. Scale bars: Left column 250 µm. Right column 50 µm. The identical symbol on different bars indicates significant differences.

The histological analysis of the femurs showed evident changes in the structure and microarchitecture of the bone among the different experimental groups. Although the non-OP animals showed a normal bone structure, in the different OP models (DEX, OVX and OD), structural changes were observed, both at the level of cortical and cancellous bone, showing a less compact bone and with a more porous structure (Figure 7a). The histomorphometric analysis revealed differences in the parameters measured in cancellous bone (Tb.N, Tb.Wi., and Tb.Sp.), with a significant reduction in all of them, in OVX and OD compared to the non-OP and DEX animals (Figure 7b–d). The cortical bone thickness (Ct.Wi.), although it showed a slight reduction in the groups (OVX and OD) with respect to the non-OP and DEX groups, was not significant (Figure 7e).

#### 3.4.2. Calvarial Critical Size Defect

**Figure 8:**
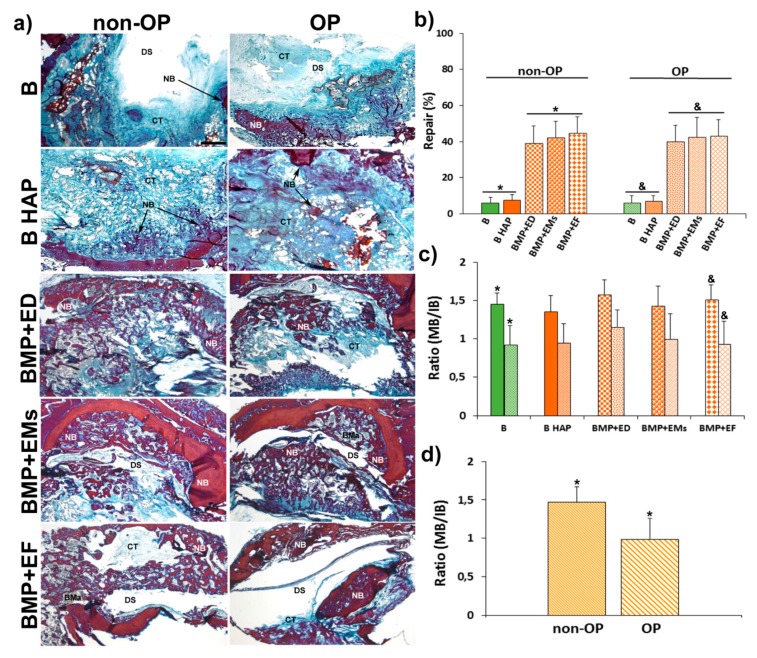
Repair process in calvarial defect. (**a**) Representative images in horizontal section of calvarial critical size defects in non-OP and OP rats showing the repair response at the defect level in the different experimental groups 12 weeks post-implantation. (**b**) Histomorphometrical analysis comparing of the degrees of repair (%) among the different experimental groups in non-OP and OP rats 12 weeks post-implantation. (**c**) Histomorphometric analysis showing the ratio between mature bone and immature bone (MB/IB) among the different experimental groups (**d**) and between non-OP and OP rats, estimated using VOF staining. Bars represent means ± SD (*n* = 4). The identical letter on different bars indicates significant differences. BMa: bone marrow; CT: connective tissue; NB: newly formed bone; DS: defect site. Scale bar = 1 mm. The identical symbol on different bars indicates significant differences.

The histological analysis at the level of the calvarial defect showed a few new bone formations in the blank groups (B and B HAP), being limited to the margins of the defect in both, non-OP, and OP groups (Figure 8a). The groups implanted with BMP-2 + 17β-estradiol in the three different formulations showed a greater area of newly formed bone in the defect area, not only in the margins, but also in inner zone of the defect (Figure 8a). The newly formed bone in the different experimental groups of non-OP and OP animals showed a normal morphology and VOF staining, revealing significant areas of mineralization, slightly higher in the groups of non-OP animals (Figure 8a).

The histomorphometric analysis showed little repair response in the blanks groups (B and B HAP) of non-OP and OP animals, with repair percent between 6 and 8%. The groups implanted with BMP-2 + 17β-estradiol in the three different formulations, on the contrary, showed a significantly higher repair response of 38–45%, with no differences being observed between non- OP and OP animals (Figure 8b).

The histomorphometric analysis of mature and immature bone showed a higher quantity of mature bone, and therefore with a greater degree of mineralization, in the experimental groups of non-OP with respect to OP animals. The ratio between mature and immature bone (MB/IB) showed individually higher values in all non-OP with respect to OP groups as well as on the whole, with values of 1.47 in non-OP animals and 0.99 in OP (Figure 8c,d).

**Figure 9:**
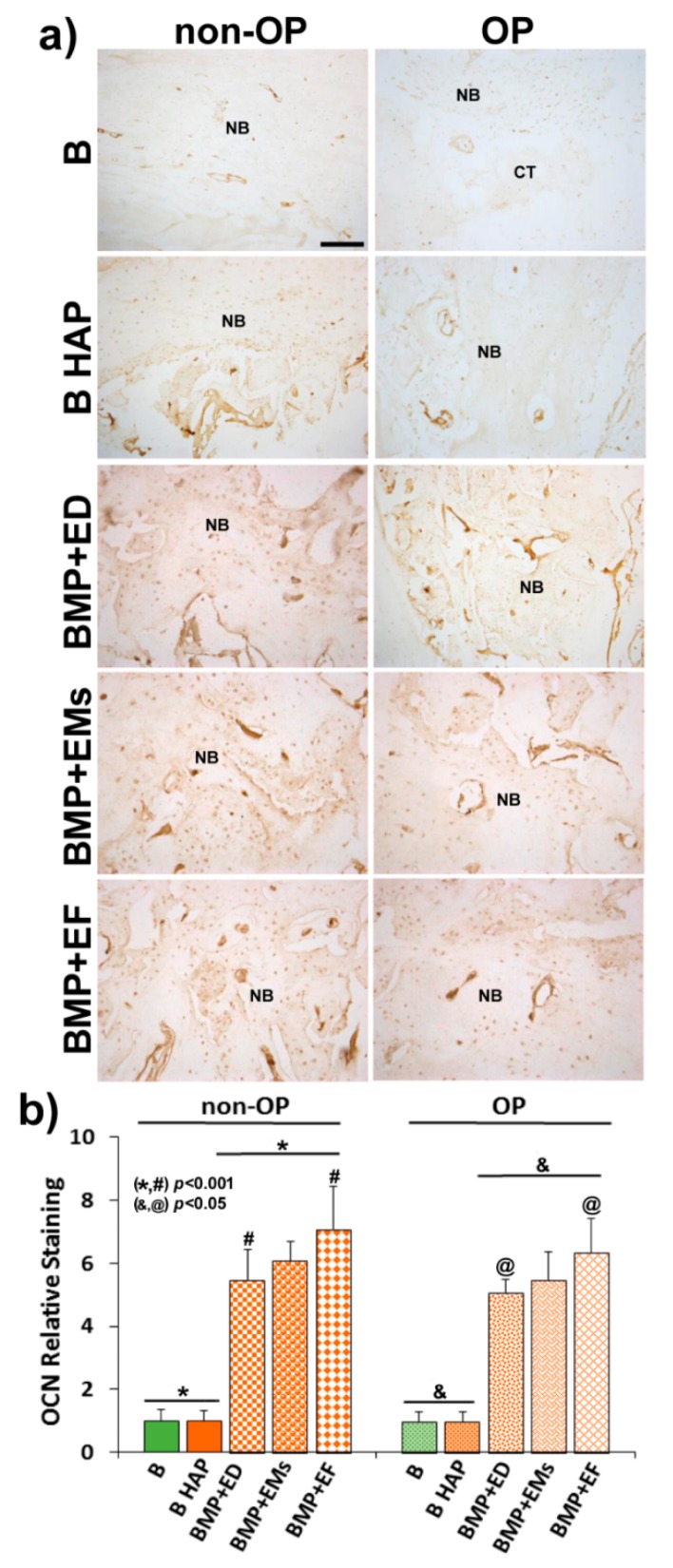
OCN relative expression. (**a**) Representative images in horizontal section of calvarial critical size defects in non-OP and OP rats showing OCN immunoreactivity in the different experimental groups 12 weeks post-implantation. (**b**) Histomorphometric analysis showing the relative staining values for OCN-ir. Bars represent means ± SD (*n* = 4). The identical letter on different bars indicates significant differences. CT: connective tissue; NB: newly formed bone. Scale bar = 100 µm. The identical symbol on different bars indicates significant differences.

The immunohistochemical analysis of osteocalcin (OCN), a marker of late osteogenesis and mineralization, showed a low immunoreaction in the blank groups (B and B HAP) in both non-OP and OP animals, with no differences between them (Figure 9a). In the groups implanted with BMP-2 + 17β-estradiol in the three different formulations, the immunoreaction was higher and more intense with respect to the blank groups, in this case being slightly higher in the non-OP animals (Figure 9a).

The histomorphometric analysis confirmed the histological data, showing slightly higher relative staining values in the BMP-2 +17β-estradiol groups in non-OP animals (Figure 9b).

## 4. Discussion

In the present study, a BMP-2-17β-estradiol hydrogel system, with porosity of approximately 72%, was evaluated for regeneration of a critical size defect in rat calvaria. Although the system had already been characterized in terms of rheological behavior, porosity, interactions between components, mass transfer parameters, and cell viability, here the good injectability of the system was showed, and its characterization has been completed by testing the water uptake and mass loss as well as differentiation studies in cultures of osteoporotic rMSCs. In vitro release profiles of 17β-estradiol in two media and the in vitro and in vivo release profile of BMP-2 were also analyzed.

The osteogenic differentiation of osteoporotic rMSCs seeded on the system was assessed in order to test the effect of the incorporation of the nano-HAP on the cell behavior. The results showed greater ALP activity and a greater number of differentiated cells in the systems with nano-HAP. Therefore, nano-HAP systems were subsequently used in the in vivo experiments.

First, a histological evaluation of the femur and calvaria of rats suffering the three treatments for osteoporosis induction was carried out. OP is a systemic bone disease characterized by the increase of bone porosity, loss of bone mass and changes in the microstructure of the skeletal. Consequently, the OP population has an increased risk of fracture.

Despite the high number of OP studies and the several publications dedicated to tissue repair in non-OP specimens, very few reports devoted to bone defect regeneration in OP have been published. As OP might be primary post-menopausal or secondary, due to corticoid chronic administration, three animal models were used for OP induction: OVX, chronic glucocorticoid treatment, and the combination of both. In previous reports, a combination OD rat model was used. However, the high deterioration observed in the animals, the risk of induced additional disorders on the skeletal and the fact that bone loss reverses after corticoid stop [33] justify the bone histological study of the different treatment, in order to simplify the model and improve animal welfare. In general, OP condition is established through the analysis of long bones and lumbar spine but few data of the effect on the calvaria of the animals used as OP models are available [34]. Most of the publications on the regeneration of calvaria critical size defect in OP animals do not report the effects of OP in the calvaria [35,36]. In the present study, the data comparing the response of the femurs and calvarias to the three treatments revealed that the effect of OVX was similar to OD combination, consequently the fabricated system was tested in OVX rats.

As some authors have observed a delay in bone consolidation of OVX rats [37] and as this combination of BMP-2 and 17β-estradiol, formulated in microspheres, when applied to a critical calvaria defect, improved bone healing in OP rats, but the new bone was less mineralized [17,18], we tried to prolong the release of active substances to cover this delay. The drugs were incorporated to the hydrogel system pre-encapsulated in microspheres for prolonged controlled release. To reduce the release rate of the active substances the microspheres were prepared with a mixture of polymers, 25% of the RG 858 was incorporated to the RG 504 for BMP-2 microspheres as well as to the RG203-S for 17β-estradiol microspheres. RG 858 is a PLGA 85:15, of high molecular weight with a degradation rate lower than that of RG 504 and RG203-S. The BMP-2 release profiles showed a two-phase behavior with a weak burst effect that coincides with the period in which the system loses mass and uptakes a high amount of water. However, the burst effect of BMP-2 that can be seen from the microspheres was damped by the hydrogel, probably due to the interaction of the protein with the chemical groups of the HAP [6,13]. Afterwards, the second phases were practically parallel, which indicated a mass transfer process controlled by the access of water inside the microspheres, dissolution and diffusion of the protein throughout the porous of the polymeric matrix.

The release profile of 17β-estradiol as liposoluble drug in a MeOH:water (60:40) release medium was previously characterized [21]. By contrast, here two release media were used, MeOH:water (50:50) and an aqueous solution of SLS, because we suspected that the solvent affected the release rate of the lipophilic substance. In fact, the release of 17β-estradiol in MeOH:water was fast regardless of the formulations. However, the in vitro release of 17β-estradiol was formulation-dependent when an aqueous medium was used; a high decrease of the release rate of the drug from the microspheres was observed. As DSC results indicated, 17β-estradiol formed a solid solution in the microspheres, which indicates that the release process takes place by molecular diffusion of 17β-estradiol within the microspheres, governed by the partition coefficient, and consequently the aqueous medium dissolved it very slowly. In addition, the higher estimated values of *D_eff_* in the MeOH:water compared with the aqueous medium, confirmed the dependency on the release media. The calorimetric analysis of the electrospun sheet was not carried out because the amount of 17β-estradiol would have had to be increased to be detected and its characteristics would have been modified. Although 17β-estradiol is expected to also be dissolved in the polymer of the film, the large specific surface area that microfibers expose to the medium causes the drug to release rapidly. Similarly, 17β-estradiol dispersed in the hydrogel was very little retained. Obviously, neither media are physiological, but it seems more correct to use an aqueous medium to predict release in vivo. Although, with reservations, one would also expect a slightly faster release rate in vivo as the biological components present in the tissue could accelerate the drug release from the system.

Despite the beneficial role of the nano-HAP controlling the BMP-2 burst effect as well as its positive effect on the proliferation and osteogenic differentiation of rMSC, which justifies the use of nano-HAP in the system, the reparative effect of the blank scaffolds with and without nano-HAP was not enough to be considered useful. Unlike that observed in this study, other authors showed better bone repair in different bone defects practiced in osteoporotic goats implanted with a system of type I collagen containing nano-HAP than without [38]. By contrast, another study [6] found, as in the present study, that the use of nano-HAP and calcium sulfate bone substitute scaffolds in rat critical calvaria defect showed no effect on repair and mineralization at 8 and 12 weeks with respect to the empty defect. In both studies, the systems were loaded with BMP-2 combined with 17β-estradiol or zolendronic acid [6] that might abolish the effect of the HAP observed in vitro.

Although previously our group reported [17,18] a better result in bone regeneration of OP animals combining BMP-2 and 17β-estradiol, in this study, the repair effect observed has been similar to that observed in non-OP groups. The three combinations of BMP-2 with 17β-estradiol in each of the three formulations used showed the same effect at 12 weeks. However, the ratios of mature and immature bone in normal and osteoporotic animals showed significant differences, indicating that the quality of the repaired bone, at least after 12 weeks, was better in normal animals.

Although these results coincide with previous work, in the present study it seems that the mineralization of the bone formed slightly improved in the OP animals. The difference in the relative osteocalcin expression was not statistically significant. These results might suggest that a longer release of BMP-2 together with the composition of the system, presence of collagen and nanoHAP favor the mineralization process. In any case, it would be necessary in future to conduct studies aimed at discerning the role of each of these components in the process. However, we have not been able to reproduce the positive effect of 17β-estradiol combined with BMP-2 in a hydrogel composed of Pluronic, Tetronic and, cyclodextrin, with other scaffolds of different composition [18]. In addition, according to the present study, the fact that the different release profiles of 17β-estradiol had no effect on the repair of the defect indicates that 17β-estradiol, when applied locally, and regardless of the release rate (available dose) and of the obvious role that it plays on bone remodeling, does not justify its inclusion as active substance in the repair of bone defects neither in normal animals nor in osteoporotic ones. Therefore, new strategies and alternative drugs are currently being designed trying to accelerate mineralization of new bone in OP groups.

## 5. Conclusions

The prepared hydrogel system resulted to be easily injectable and solidified fast due to crosslinking of collagen and chitosan chains. The system helps control the burst effect of BMP-2 pre-encapsulated in PLGA microspheres, probably due to the nano-HAP. Release of 17β-estradiol from PLA-PLGA microspheres was more complex and is governed by the partition coefficient of the drug which is in solid dissolution in the microspheres. The system was biocompatible both in vivo and in vitro. However, the regenerative effect detected in the critical bone defect of both OP and non -OP rats was mainly due to the osteogenic effect of BMP-2 released in a controlled rate for 6 weeks. A delay in the mineralization of the new bone which fills the defect in OP animals was observed. 17β-estradiol released from different formulations and included in the system does not improve bone repair.

## Figures and Tables

**Table 1:** Experimental groups to evaluate regenerative efficiency.

Group Designations	Treatment
Blank I (B)	System loaded with blank microspheres and blank films
Blank II (B-HAP)	System loaded with blank microspheres and 5 mg of nano-HAP and blank films
BMP+EF	System loaded with 6 µg of BMP-2 in microspheres and 200 µg of 17β-estradiol in the 2 films
BMP+EMs	System loaded with 6 µg of BMP-2 in microspheres and 200 µg of 17β-estradiol in microspheres, blank films
BMP+ED	System loaded with 6 µg of BMP-2 and 200 µg of 17β-estradiol dispersed, blank films

**Table 2:** Characteristics of the component of the sandwich-like system. Microspheres: size and encapsulation efficiency. Electrospun film: thickness and porosity of the film and average diameter of the fibers). Core system: porosity freshly prepared and lyophilized and water uptake and mass loss after incubation in MilliQ water, 37 °C, and 25 rpm.

Microspheres	Size (µm)			E.E. (%)	
17β-estradiol	101.4 (10% < 29.73, 90% < 198.31)			83.5 ± 1.84	
BMP-2	112.1 (105 < 60.3, 905 < 174.8)			71 ± 7	
Film	Thickness (µm)	Fiber diameter (µm)			Porosity (%)
	63.4 ± 4.3	1.2 ± 0.26			71.9 ± 0.41
Core system (Hydrogel + microspheres)	Porosity (%)	Incubation time	Water uptake (%)		Mass loss (%)
	72	7 days	135.9 ± 2.6		18.85 ± 7
		28 days	138.9 ± 11.0		29.19 ± 4.88

**Table 3:** Estimated values of effective diffusion coefficient (*D_eff_*) and mass transfer coefficient (*h*) for 17β-estradiol release in different media applying Equations (3)–(6).

Release Media	*D_eff_* (m^2^/s)	*h* (m/s)	*R*^2^ (%) Value
MeOH:water (50:50)	2.28·10^−15^ ± 5.00·10^−17^	7.56·10^−10^ ± 2.89·10^−10^	95.47 ± 0.07
SLS 1%	5.58·10^−16^ ± 9.81·10^−17^	4.01·10^−10^ ± 4.94·10^−10^	92.49 ± 2.71
